# Physical Feature Encoding and Word Recognition Abilities Are Altered in Children with Intractable Epilepsy: Preliminary Neuromagnetic Evidence

**DOI:** 10.1155/2015/237436

**Published:** 2015-06-03

**Authors:** Maria Pardos, Milena Korostenskaja, Jing Xiang, Hisako Fujiwara, Ki H. Lee, Paul S. Horn, Anna Byars, Jennifer Vannest, Yingying Wang, Nat Hemasilpin, Douglas F. Rose

**Affiliations:** ^1^Division of Neurology, Cincinnati Children's Hospital Medical Center, Cincinnati, OH 45229, USA; ^2^Milena's Functional Brain Mapping and Brain-Computer Interface Lab, Florida Hospital for Children, Orlando, FL 32803, USA; ^3^Comprehensive Pediatric Epilepsy Center, Florida Hospital for Children, Orlando, FL 32803, USA; ^4^MEG Lab, Florida Hospital for Children, Orlando, FL 32803, USA; ^5^Division of Biostatistics & Epidemiology, Cincinnati Children's Hospital Medical Center, Cincinnati, OH 45299, USA; ^6^Pediatric Neuroimaging Research Consortium, Cincinnati Children's Hospital Research Foundation and Cincinnati Children's Hospital Medical Center, Cincinnati, OH 45229, USA; ^7^Biomedical Engineering, University of Cincinnati, Cincinnati, OH 45221, USA

## Abstract

Objective evaluation of language function is critical for children with intractable epilepsy under consideration for epilepsy surgery. The purpose of this preliminary study was to evaluate word recognition in children with intractable epilepsy by using magnetoencephalography (MEG). Ten children with intractable epilepsy (M/F 6/4, mean ± SD 13.4 ± 2.2 years) were matched on age and sex to healthy controls. Common nouns were presented simultaneously from visual and auditory sensory inputs in “match” and “mismatch” conditions. Neuromagnetic responses M1, M2, M3, M4, and M5 with latencies of ~100 ms, ~150 ms, ~250 ms, ~350 ms, and ~450 ms, respectively, elicited during the “match” condition were identified. Compared to healthy children, epilepsy patients had both significantly delayed latency of the M1 and reduced amplitudes of M3 and M5 responses. These results provide neurophysiologic evidence of altered word recognition in children with intractable epilepsy.

## 1. Introduction

Progression of epilepsy may negatively affect language and other higher order cognitive functions, especially in young children. Impairments of receptive language function (language comprehension) in epilepsy patients are demonstrated by problems in phonological processing [1] and reading [2, 3], which requires integration of fundamental cognitive functions, visuospatial capacities, and attention, as well as long- and short-term memory [2].

Approximately 20–30% of children with epilepsy continue to have disabling seizures despite high-dose medications (intractable epilepsy) or develop intolerable medication side effects. For some of those children, surgical intervention is undertaken to ameliorate and often cure the epilepsy [4]. For this particular group of children, evaluation of their language function (and its neural correlates in particular) is important and may contribute to surgical decision-making [5].

Magnetoencephalography (MEG) is a diagnostic tool for evaluating patients with medically refractory epilepsy and localizing epilepsy focus [6]. It also allows for the assessment of neural substrates involved in receptive language function. MEG is a noninvasive neurophysiological technique with high temporal resolution, which can provide information about the neural events related to a variety of aspects of language processing, including single word reading [7–9]. Moreover, event-related fields (ERFs), defined as time-locked changes to external stimuli (words, phrases, and sentences) in MEG activity, can provide an objective index of neurophysiological processing associated with language function in the human brain.

MEG studies focusing on visual word recognition have been used in patients with intractable epilepsy to examine language processing and language lateralization [10]. Furthermore, MEG tests of recognition of acoustically presented words have been used for identifying atypical receptive language function in epilepsy patients [11]. This is the first MEG study in epilepsy patients that used word presentation in both visual and auditory modalities. It is worth mentioning that simultaneous auditory and visual presentation happens frequently in activities of daily living. Results of recent fMRI studies suggest that simultaneous audio-visual presentation of sounds and images of three-dimensional objects shortens processing times in early sensory cortices, thus allowing faster information processing of surrounding environmental changes [11]. Previously, we described a paradigm that uses simultaneous visual and auditory word presentation for the testing of receptive language function in a healthy pediatric population [12].

The objective of this study was to use this previously developed paradigm of simultaneous auditory and visual word presentation [12] to explore neuromagnetic brain activity associated with word recognition in children and adolescents with intractable epilepsy. We chose this paradigm because simultaneous presentation of the written word (visual information) and its acoustic analogue (auditory information) (1) allows for the exploration of audiovisual processing; (2) potentially maximizes neural involvement (activation) from brain structures responsible for word processing [13, 14]; and (3) potentially may shorten language processing/comprehension time [11].

## 2. Methods

The study was approved by the Cincinnati Children's Hospital Medical Center (CCHMC) institutional review board.

### 2.1. Participants

Ten children with medically intractable epilepsy that were undergoing evaluation for epilepsy surgery were matched with a single healthy control by age (within one year) and sex. There were 4 females and 6 males in each group. The primary language for the subjects in both groups was English.

The age range of the epilepsy group was 10–17 years with a mean of 13.4 ± 2.2. All epilepsy subjects were right-handed and additional characteristics are summarized in Table 1. Their data was collected after their routine clinical MEG evaluation for preoperative functional localization was completed at the CCHMC epilepsy surgery program.

The age range of the healthy controls was 9–17 years with a mean of 13.5 ± 2.4. The controls assented and informed consent was obtained from their parents. The controls were right-handed, as measured by the Edinburgh Handedness Inventory [15], and had normal or corrected-to-normal vision and normal hearing. The controls had no history of neurological or psychiatric disorders and were not on medication.

### 2.2. Clinical Evaluation

All patients underwent presurgical evaluation in order to determine the epileptogenic area. The evaluation included seizure characterization by clinical semiology, video-EEG (VEEG) including overnight recording while the patients slept, magnetic resonance imaging (MRI) with epilepsy surgery protocol, ictal/interictal single photon emission computed tomography (SPECT), 2-deoxy-2[^18^F]fluoro-D-glucose positron emission tomography (FDG-PET), simultaneous MEG and EEG, and functional MRI (fMRI). None of the patients had electrographic status epilepticus of slow wave sleep (ESES). None of the patients had been previously diagnosed as Landau Kleffner nor did any of the patients fit the clinical profile for Landau Kleffner. The results of simultaneous MEG/EEG recording provided information about average spike frequency for each patient (Table 1).

#### 2.2.1. Clinical Outcome

A total of 10 patients finished the full process of presurgical evaluation. Of those 10 patients, 8 patients underwent resective surgery. Seven patients (Engel Class I, 88%, 7/8) were seizure-free and 1 patient (Engel Class II, 12%, 1/8) had rare seizures after mean follow-up duration of 26 months (range 15–47 months). The pathology showed a focal cortical dysplasia in 7 patients and an ischemic change in 1 patient. Two patients did not have surgery because of incomplete evaluation or discordant test results. None of the patients experienced appreciable neurological deficits as a result of surgery.

#### 2.2.2. Intellectual and Academic Assessment

The routine neuropsychological examination of epilepsy surgery candidates included the Wechsler Intelligence Scale for Children, Fourth Edition (WISC-IV) [16]. The Wechsler scales are the most widely used measures of intelligence and have excellent reliability and validity. The WISC-IV yields a Full Scale IQ (FSIQ) score as well as four factor scores: Verbal Comprehension Index (VCI), Perceptual Reasoning Index (PRI), Processing Speed Index (PSI), and Working Memory Index (WMI). Selected subtests of the Woodcock-Johnson III Tests of Achievement (WJ-III) [17] were also administered. Those subtests included Letter-Word Identification, which measures single word reading, and Passage Comprehension, which measures reading comprehension.

### 2.3. Stimuli

**Figure 1:**
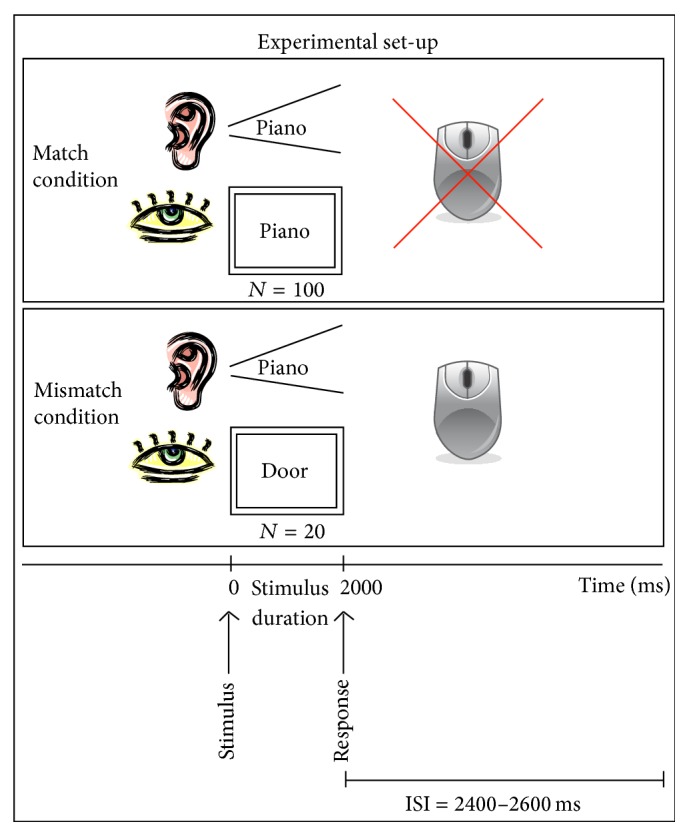
Visual representation of the audio-visual word presentation paradigm. Stimuli were presented simultaneously from visual (screen) and auditory (earphones) sensory inputs in two different condition (1) “match” condition, for which the visually and acoustically presented words were identical, and (2) “mismatch” condition, for which the visually and acoustically presented words were different. The participants were asked to compare visually and acoustically presented words and to press the response button only if they did not match.

The stimuli consisted of 120 common nouns that were one to three syllables (mean 1.35 syllables) and three to eight letters (mean 4.88 letters) based on counts of Kucera et al. [18] in the MRC Psycholinguistic Database [19]. We selected relatively short, highly frequent words that would likely be read accurately by study participants.  Figure 1 illustrates the paradigm we used. Spoken and written words were presented simultaneously. There were two conditions in this paradigm: (1) “match” condition, for which the visually and acoustically presented words were identical (*N* of words = 100), and (2) “mismatch” condition, for which the visually and acoustically presented words were different (*N* of words = 20). The participants were asked to compare the visually and acoustically presented words and to press a response button if the spoken and written words did not match. The “mismatch” condition with required motor response was included only to verify that the subject continued to attend to the task throughout the study. Each word presentation duration was 2,000 ms. The interstimulus interval was randomized between 1200 and 1400 ms to avoid prediction of the stimulus onset by the subjects. The visual word stimuli were projected on a screen as white letters on a black background [20, 21]. The average distance between the screen and the nasion of the subject was 350 mm and the average visual angle was 3.27 degrees. For the acoustical presentation, the words were delivered through plastic tubes into subject's left and right ears with averaged intensity of 75 dB nHL. BrainX software (developed by study coauthor Dr. Jing Xiang) was used for stimulus delivery [22, 23].

### 2.4. Data Recording

A standard protocol for data acquisition as described in our previous studies [22, 23] was used. MEG signals were recorded with a 275-channel whole head MEG system (VSM MedTech Ltd., Port Coquitlam, BC, Canada) in a magnetically shielded room (Vacuum-Schmelze, Hanau, Germany). The recording sessions required each participant to lie as still as possible on a bed with his or her head inside the MEG helmet for approximately 8 min for the performance of the paradigm. All participants laid in the supine position with their arms rested on either side. They were instructed to avoid eye blinks and head movements. Before data acquisition started, 3 electromagnetic coils were placed on the nasion, at the left, and right preauricular points of each subject. The coils were used to measure the position of the sensor array with respect to the nasion-ear coordinate system in order to track head motion. Data were recorded at a sampling rate of 6,000 Hz with a noise cancellation of third order gradients. The acquisition window was 2,600 ms, beginning 600 ms before each word presentation.

### 2.5. Data Processing and Analysis

MEG data were corrected with DC offsets based on the pretrigger time-window. An off-line low pass filter (30 Hz) and high pass filter (3 Hz) were applied to the averaged MEG data. The analysis window was 600 ms before the stimuli and 2,000 ms after the stimuli. This study focused on the responses from the “match” condition, as the “mismatch” condition was used only to ensure subjects' attention to the stimuli. The latencies and the peak amplitudes of averaged MEG waveform were measured for each recognizable component with the DataEditor software (VSM MedTech Ltd., Port Coquitlam, BC, Canada). There were five major and consistent peaks, labeled M1–M5 at latencies of ~100 ms (50–120 ms), ~150 ms (150–200 ms), ~250 ms (250–300 ms), ~350 ms (300–400 ms), and ~450 ms (400–500 ms), respectively. The first of these peaks (M1 and M2) are usually described as representing processing of stimuli characteristics (e.g., encoding of physical stimuli features) separately for each sensory modality [8], whereas later components (M3–M5) are associated with more complex processing, requiring integration of information from both visual and auditory modalities together [24, 25]. Some peaks had multiple components within their time range; in this case, the peak latency was determined at the point of the highest amplitude in the defined range. In order to analyze left and right responses individually, the MEG channels were separated into left and right hemisphere groups.

### 2.6. Statistical Analysis

#### 2.6.1. Between-Group Comparison

Study results were analyzed with SAS software version 9.1 (SAS Institute, Cary, NC). The amplitude and latency comparisons for ERF components M1, M2, M3, M4, and M5 were conducted with a mixed model analysis of variance (ANOVA) with two fixed factors:* GROUP*: epilepsy patients* versus* healthy controls and* HEMISPHERE*: left* versus* right and one random factor:* SUBJECT* nested within group. Statistically significant effects were tested for multiple ERF comparisons using a False Discovery Rate (FDR) procedure [26].

#### 2.6.2. Correlation Analysis

In order to estimate the relationship between neurophysiological changes and disease characteristics, the amplitudes and latencies of the ERF components were correlated (1) with the age of epilepsy patients at disease onset (“age at epilepsy onset”) and (2) with the duration of disease (“epilepsy duration”). In order to detect possible relations between IQ and neurophysiological parameters in epilepsy patients, the IQ scores (VCI, PRI, WMI, PSI, and FSIQ), reading and language comprehension scores (WL L-W, WJ Pcomp), and the ERF parameters were analyzed (latencies and amplitudes separately at left and right hemispheres). In order to investigate the effect of spike frequency on ERFs, average spike frequency during the recording of word “match” condition was correlated with ERFs parameters (latencies, amplitudes). All correlations were calculated with Spearman's rho.

## 3. Results

### 3.1. Neurophysiology and Correlations

**Figure 2:**
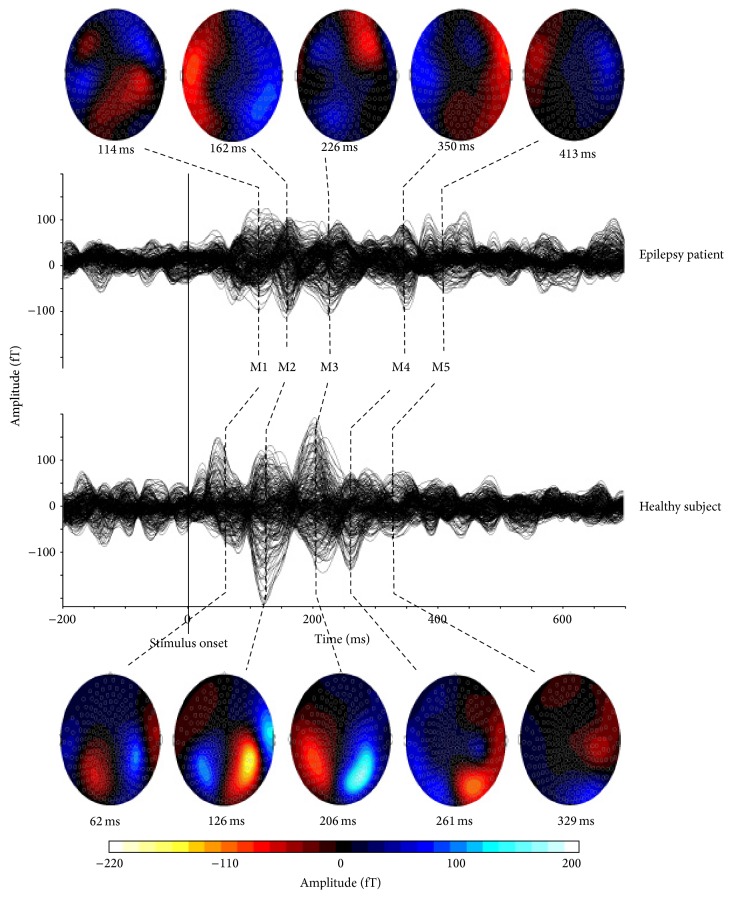
MEG waveform and topographical map of neuromagnetic activation elicited by visual and auditory words. Five major neuromagnetic responses are clearly identified. They are named as M1, M2, M3, M4, and M5. In topographical maps, red color represents the incoming magnetic fields; blue color represents outgoing magnetic fields. Epilepsy patient (top) had delayed latencies of M1 and M2 components as compared with the healthy control subject (bottom). The amplitudes of M3 and M5 magnetic fields were significantly smaller in epilepsy patients than in healthy controls.

The latencies of M1 and M2 components were delayed in both hemispheres in epilepsy patients compared to healthy controls (Table 2, Figure 2). The main effect of* GROUP* was significant for M1 component with *F*(1, 18) = 8.83, *p* = 0.008, as well as for M2 component with *F*(1, 18) = 4.46, *p* = 0.049. Moreover, the amplitudes of M3 and M5 components were smaller in both hemispheres in epilepsy patients than in healthy subjects (Table 3). The main effect of* GROUP* was significant for M3 component with *F*(1, 18) = 7.91, *p* = 0.012 and for M5 component *F*(1, 18) = 7.04, *p* = 0.016. After applying the FDR procedure for multiple comparisons, the main effect of group on M1 latency and on M3, M5 amplitudes remained significant. There were no significant main effects of* HEMISPHERE* on either ERF latencies or amplitudes and no significant* GROUP* ×* HEMISPHERE* interaction.

There was no significant correlation between spike frequency and ERFs parameters.

### 3.2. Neuropsychological Scores and Correlations

Patients' IQ scores ranged from average to mildly impaired with overall group performance score in the borderline range (see Table 1). Seven of eight patients had borderline to mildly impaired WMI and PSI scores. Word reading and Reading Comprehension subtest scores ranged from mildly impaired to average.

**Figure 3:**
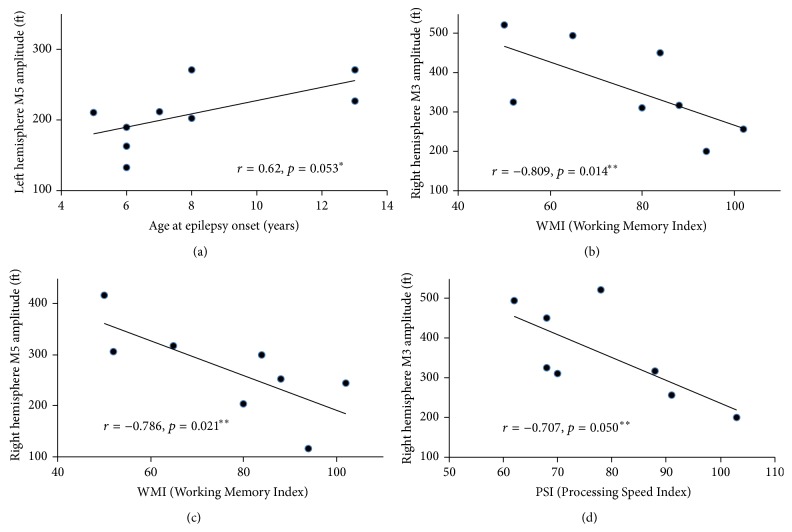
Graphic representation of correlation analysis results: (a) relationship between left hemisphere M5 component amplitude and age at epilepsy onset; (b) relationship between right hemisphere M3 component amplitude and Working Memory Index (WMI); (c) relationship between right hemisphere M5 component amplitude and WMI; (d) relationship between right hemisphere M3 component amplitude and Processing Speed Index (PSI). “*∗*” is indicated trend toward statistical significance; “*∗∗*” are indicated statistically significant results before applying False Discovery Rate (FDR) procedure for multiple comparisons.

There was a trend toward significant positive correlation (*r*_*s*_ = 0.62, *p* = 0.053) between the left hemisphere M5 amplitude and age at epilepsy onset (Figure 3(a)). Correlation analyses between the ERF parameters (M1 latency, M3 and M5 amplitudes) and the neuropsychological measures in epilepsy patients revealed significant negative correlation between the M3 amplitude in the right hemisphere and the WMI (*r*_*s*_ = −0.809; *p* = 0.014) (Figure 3(b)), between M5 amplitude in the right hemisphere and the WMI (*r*_*s*_ = −0.786; *p* = 0.021) (Figure 3(c)), as well as between M3 amplitude in the right hemisphere and the PSI (*r*_*s*_ = −0.707; *p* = 0.050) (Figure 3(d)). However, these values did not remain significant after applying the FDR procedure for multiple comparisons. There were no significant correlations between ERFs parameters and WJ-III (single word reading and comprehension).

## 4. Discussion

### 4.1. Identified ERF Responses

Neuromagnetic responses M1–M4 identified in this study are comparable with those we found in the healthy adult population [12]. We described four major components M1–M4 with latencies around 100 ms, 150 ms, 250 ms, and 350 ms. Besides, we defined an additional M5 component peaking around 400–450 ms.

Neuromagnetic responses to word stimuli can be separated into two groups: early (M1 and M2 components) and late (components M3–M5) [27]. Early components are thought to originate from primary sensory areas. In this way, visually presented words activate primary visual cortex, whereas the same words presented acoustically activate primary auditory cortex as reflected in early neuromagnetic responses at latencies earlier than 150 ms [8]. Late MEG responses occur between 250 and 450 ms after stimulus onset and are considered to be language-specific in language task paradigms and require convergence from both auditory and visual inputs [24, 25]. According to Halgren et al. [27], they originate in Wernicke's area and through anterior temporal sites spread to Broca's area, and then further to anterior orbital, perisylvian, frontopolar, and dorsolateral prefrontal regions.

There were no significant differences between the right and left hemisphere latencies or amplitudes. This implies that the utilized word recognition task produced bilateral language activation and cannot be recommended at its present form as a task for determining language dominance. Future studies focusing on specific components elicited during task presentation combined with source analysis of electromagnetic brain activity are advised to investigate possible task application for identifying hemispheric dominance for language.

### 4.2. Latency Delay of M1 Component in Epilepsy Patients

In our study, the latency of M1 was significantly delayed in epilepsy patients as compared with the group of healthy control participants. M1 belongs to a group of early MEG responses, which are thought to reflect activation of the primary auditory and visual sensory cortices [8]. In the auditory modality, M1 is the first prominent component (also referred to as N100 m or M100 in MEG and N1 or N100 in EEG), spearing about 100 ms after the stimulus onset. M1 reflects the encoding of physical and early temporal stimulus features [28]. It is localized in auditory cortex on the posterior surface of the superior temporal gyrus [29, 30]. In the visual modality, during visual word recognition tasks, this early response (<200 ms) was localized using single equivalent current dipole (ECD) in caudal and mesial occipital regions (primary and association visual cortices) [31]. Our finding of a delay in M1 latency in intractable epilepsy patients compared to healthy controls is in line with previous studies of auditory information processing [32, 33], demonstrating that the delayed analogue of the magnetic M1 response in EEG (N100 response) was associated with the spikes in the primary auditory cortex in patients with focal epilepsy. Moreover, another EEG study, investigating auditory processing in the Landau-Kleffner syndrome, showed that the left hemisphere paroxysmal activity was associated with longer latency and lower auditory evoked potentials (AEPs) amplitudes than right hemisphere, and both right and left temporal AEPs had longer latency and lower amplitude than healthy controls [34]. Finally, the current results are in line with our recent finding that, compared with healthy controls, children with intractable epilepsy had significantly reduced M100 amplitude, which was interpreted as a reduction in neuronal resources participating in auditory information processing [22]. The delayed M1 latency of intractable epilepsy patients in our current study could represent the delay in the conduction times of auditory and visual information processing within the primary sensory cortical areas. Our findings suggest that in children with intractable seizures, the delay in the time course of word recognition is present early, at the level of auditory and visual physical stimulus feature encoding.

### 4.3. Amplitude Reduction of M3 and M5 Components in Epilepsy

We observed reduced amplitudes of M3 and M5 components in patients with intractable epilepsy when compared with healthy controls. Both M3 and M5 components belong to a group of late MEG responses, peaking between 250 and 450 ms after stimulus onset, respectively. These responses follow primary cortex activations (reflected in responses M1 and M2) and represent phonological and semantic processing, engaging inferior frontal gyrus (Brocas's area), superior/middle temporal gyrus, and angular/supramarginal gyrus (Wernicke's area) [9, 35]. Previous MEG language studies have shown that neuromagnetic signals with latencies between 250 and 450 ms (M3–M5) are language-specific in language task paradigms and may be related to particular features of linguistic stimuli, such as duration, frequency, and semantic information [24, 25, 27]. Responses in the visual modality peaking between 200 and 600 msec after stimulus onset and related to word recognition were interpreted to reflect receptive language function [31].

The association between visual and auditory word recognition was confirmed in a number of MEG studies by Salmelin [9]. Other studies, investigating the interaction between auditory (phonetic) and visual (graphemic) inputs with single letters, showed that their first convergence occurs around 225–280 ms after stimulus onset and may be conceptualized as the merging of auditory and visual streams [36]. As a result of this convergence and interaction between auditory and visual inputs, the phoneme and grapheme are integrated [36]. Specifically, this integration of visual and auditory sensory information streams during word recognition involves the left posterior superior temporal gyrus (STG) [37]. In addition, M3–M5 responses, when elicited to both aurally and visually presented words, were localized in the posterior temporal region [8]. Therefore, M3 (250 ms) and M5 (450 ms) amplitude reduction could imply that our patients had reduced neural resources for initial integration of visual and auditory inputs (M3) and comprehension (M5) of information associated with word processing and recognition. These functional abnormalities can potentially occur at STG and superior parietal lobule. Similar to other neuroimaging studies, we found functional abnormalities in language processing in epilepsy patients compared to healthy controls, as reflected in reduced BOLD fMRI activation to language stimuli and delayed MEG auditory evoked field latencies [38].

These results could have implications for epilepsy patients with regard to intervention options aimed at improving word recognition and language comprehension in general. Visual stimulation has been reported to produce changes in auditory brain response, depending on whether the visual stimulus is congruent (match) or noncongruent (mismatch). If the auditory and visual stimuli are congruent, the response to auditory stimuli (or participation of neuronal resources in it) increases [37]. Therefore, in patients with reduced M3 and M5 responses, the amplitude of both these components could be potentially increased by the visual input during the word presentation, which in turn may improve reading performance. Moreover, overall speed of processing can be facilitated by simultaneous audiovisual stimulation, as was shown by Fuhrman Alpert et al. [11], who found that latencies of brain activity at primary auditory and visual cortices are shorter for audio-visual stimulation (such as simultaneous object's word and picture presentation) compared with those for auditory or visual stimulation alone. The beneficial effect of bimodal presentation on recruitment of neuronal resources was confirmed in a recent MEG study by Jenkins et al. [13]. They showed that bimodal stimulation (such as simultaneous presentation of auditory signal “pseudo-speech” and similarly modulated visual signal “pseudo-mouth”), when compared to unimodal stimulation, produced greater response power, and as a consequence, greater neuronal involvement.

### 4.4. Effect of Epilepsy on Brain Neuromagnetic Activity

There was a strong trend for a positive correlation between M5 amplitude and the age at epilepsy onset; this means that earlier age at epilepsy onset was associated with smaller M5 amplitudes. This suggests that early epilepsy onset has a negative impact on word processing (integration of both visual and auditory modalities) and is associated with insufficient neuronal resources participating in this stage of word recognition. Our observed result is in line with our previous study [22], showing a general trend for a negative correlation between latencies of magnetic mismatch negativity response (MMNm) and age at epilepsy onset. This suggests that early epilepsy onset weakens cortical sound discrimination and processing. The relationship between age at onset and cognitive functioning was also demonstrated in temporal lobe epilepsy studies that found early onset of epilepsy can hinder development of higher order temporal lobe functions, thereby leading to lower educational levels [39, 40].

One can argue that combining the majority of left-hemispheric cases (80% of studied patients) with right-hemispheric epilepsy cases (20%) can bias the results towards stronger observed effect on language-related function. This would to certain degree apply to adult epilepsy patients. In children, however, the reorganization of language-related function occurs (specifically in those with the early-onset epilepsy) [41–43] and the effect of left-localized seizures on language-related function may not differ significantly from those with the right-hemispheric seizure focus. Future studies are needed that compare left-versus right-lateralized pediatric epilepsy groups.

We did not find any significant effect of spike frequency on ERFs amplitudes or latencies. This is consistent with previous studies [44] that found the influence of spike frequency on late ERP responses was insignificant. It was an anticipated result as the timing and duration of the spikes (if there were any) were random, whereas ERF responses are time-locked to stimuli. Therefore, our observed ERF changes in epilepsy patients when compared to healthy control subjects can be attributed with a higher degree to the pathology of epileptogenic zone itself rather than to the epileptic current. The evidence of epileptic activity per se interfering with propagation of auditory information was shown in a number of previous studies (e.g., see [45]). However, further investigations are needed in order to confirm or disprove these findings.

### 4.5. Study Limitations and Possible Effects of Drugs

The experience at epilepsy centers worldwide shows that intractable epilepsy patients are a challenging group to study. Some of the drugs used could have an inhibiting effect on ERF responses by decreasing their amplitudes and delaying their latencies through activation of the GABA system [46]. However, the majority of administered drugs have a relatively short time to peak concentration, and a short period of half-life elimination, whereas longer half-life elimination drugs are usually titrated in advance of admission for Phase I (noninvasive presurgical) testing. Since our patients had no medication for at least 12 hours prior to MEG recording, the acute effect of medication on ERF parameters can be considered non severe. However, the possible long-term effect of antiepileptic medication on ERFs cannot be excluded [47]. This question must be addressed in future studies. The study is also limited due to the small sample size. We are planning to analyze MMNm source locations in a future, larger scale study.

Because the main purpose of this study was to demonstrate the application of a novel paradigm for evaluation of word recognition in children with intractable epilepsy, we did not focus on matching subjects to controls by IQ. However, this is an important issue that needs to be addressed in future studies. For example, to better answer the question whether epilepsy itself or the altered neural substrate leads to impairment in information processing/cognitive functioning, future studies should match the borderline IQ of epilepsy patients to control subjects without epilepsy.

## 5. Conclusions

In conclusion, the results of our preliminary study were as follows: when compared to healthy subjects, patients with intractable epilepsy had (1) delayed conduction times of encoding of both visual and auditory stimuli features (reflected in M1 latency delay) and (2) reduced neuronal resources required for integration of audio and visual streams, required for performing this word recognition task (reflected in M3 and M5 amplitude reduction). The effect of interventions can be studied with this paradigm by recording MEG before and after intervention. To our knowledge, this is the first study that described simultaneous written and spoken word stimuli presentation in pediatric epilepsy patients. A larger scale study, based on described methodology and focusing on cortical generators of registered magnetic activity, would further our understanding of neural origins of language comprehension in children with intractable epilepsy.

## Figures and Tables

**Table 1:** Characteristics of patients.

Nr.	Demographics			Diagnosis	Side	Epilepsy onset, years	Epilepsy duration years	Pathology	Average spike frequency^b^	Antiepileptic drugs^c^	Neuropsychological exam							Seizure outcome	F/U duration (mo)
	Age, y	Sex	DH	Epilepsy focus^a^							VCI	PRI	WMI	PSI	FSIQ	WJ L-W	WJ Pcomp	Engel's class	
1	13	F	R	Temporal	L	6	7	FCD 2A	17	Lamotrigine, topiramate, clonazepam	79	94	94	103	88	79	76	Class 1	24

2	14	F	R	Frontal, temporal	L	6	8	FCD 1B	33	Levetiracetam, ethosuximide, methosuximide	67	82	52	68	60	65	75	Class 1	28

3	15	M	R	Frontal	L	13	2	FCD 1A	38	Gabapentin, divalproex sodium	93	102	80	70	84	113	104	Class 1	26

4	12	M	R	Occipital, temporal	L	7	7	old ische-mic change	46	Levetiracetam, oxcarbazepine, zonisamide	87	67	65	62	65	101	91	Class 1	36

5	12	M	R	Frontal	R	8	4	FCD 2A	0	Topiramate, lamotrigine	71	94	50	78	62	87	67	Class 1	19

6	11	F	R	Temporal^*∗*^, parietal	L	6	5	FCD 2B	100	Levetiracetam, lamotrigine, clonazepam	NT	98	88	88	85	95	99	Class 1	15

7	14	M	R	Frontal, parietal, temporal	R	13	1	NA	14	Oxcarbazepine, gabapentin	NT	NT	NT	NT	NT	89	78	No surgery yet	NA

8	10	M	R	Parietal	L	8	2	FCD 1B	0	Levetiracetam, divalproex sodium	115	102	102	91	105	107	97	Class 1	47

9	17	M	R	Temporal^*∗*^, frontal	L	5	8	FCD 1B	5	Levetiracetam	73	73	71	75	68	93	92	Class 1	19

10	16	F	R	Frontal^*∗*^, temporal	L	7	9	NA	0	Lamotrigine, levetiracetam	NT	NT	NT	NT	NT	105	96	No surgery yet	NA

M ± SD	13.4 ± 2.2					7.9 ± 2.8	5.3 ± 2.9		25.3 ± 31.2		87.4 ± 16.9	92.7 ± 12.4	76.8 ± 19.2	78.5 ± 14.2	80 ± 16.03	93.4 ± 14.3	87.5 ± 12.5		26.8 ± 10.4

Min	10					5	1		0		67	67	50	62	60	65	67		15

Max	17					13	9		100		115	103	102	103	105	113	104		47

F: female; M: male; R: right; L: left; NT: not tested; NA: not applicable; DH: Dominant Hand; FCD: focal cortical dysplasia; VCI: Verbal Comprehension Index; PRI: Perceptual Reasoning Index; PSI: Processing Speed Index; WMI: Working Memory Index; FSIQ: Full Scale IQ; WJ L-W: Letter-Word Identification; and WJ Pcomp: Passage Comprehension.

^a^A major epilepsy focus is marked with a star *∗*.

^b^Number of spikes in 40 min of MEG.

^c^Given the night before study (at least 12 h).

**Table 2:** Latencies (ms, mean ± SD) of M1–M5 components separately from left and right hemispheres in epilepsy patients and healthy controls.

	M1		M2		M3		M4		M5	
	Left	Right	Left	Right	Left	Right	Left	Right	Left	Right
Epilepsy	114.1 ± 19.9	109.74 ± 19.7	181.7 ± 34.6	185.6 ± 32.0	264.0 ± 41.3	263.6 ± 35.5	371.5 ± 51.9	371.5 ± 54.6	474.2 ± 43.8	475.4 ± 47.1

Controls	90.3 ± 13.4	92.73 ± 12.7	154.8 ± 21.7	161.7 ± 18.7	248.86 ± 28.7	255.6 ± 24.0	341.3 ± 38.4	349.4 ± 49.5	450.0 ± 54.4	447.3 ± 59.9

**Table 3:** Amplitudes (fT, mean ± SD) of M1–M5 components separately from left and right hemispheres in epilepsy patients and healthy controls.

	M1		M2		M3		M4		M5	
	Left	Right	Left	Right	Left	Right	Left	Right	Left	Right
Epilepsy	350.1 ± 150.6	317.0 ± 119.0	422.4 ± 152.6	339.1 ± 131.2	362.8 ± 111.8	377.7 ± 109.0	293.5 ± 78.4	330.4 ± 129.5	230.4 ± 82.9	266.7 ± 85.7

Controls	447.7 ± 202.0	448.3 ± 148.7	487.5 ± 147.7	477.1 ± 149.8	544.6 ± 142.7	493.3 ± 165.	421.5 ± 133.0	353.6 ± 104.2	343.4 ± 120.3	266.7 ± 85.7
